# PI3K‐α/mTOR/BRD4 inhibitor alone or in combination with other anti‐virals blocks replication of SARS‐CoV‐2 and its variants of concern including Delta and Omicron

**DOI:** 10.1002/ctm2.806

**Published:** 2022-04-07

**Authors:** Arpan Acharya, Anup S. Pathania, Kabita Pandey, Michellie Thurman, Kendra R. Vann, Tatiana G. Kutateladze, Kishore B. Challagundala, Donald L. Durden, Siddappa N. Byrareddy

**Affiliations:** ^1^ Department of Pharmacology and Experimental Neuroscience University of Nebraska Medical Center Omaha Nebraska USA; ^2^ Department of Biochemistry and Molecular Biology University of Nebraska Medical Centre Omaha Nebraska USA; ^3^ Department of Pharmacology University of Colorado School of Medicine Aurora Colorado USA; ^4^ The Child Health Research Institute University of Nebraska Medical Center Omaha Nebraska USA; ^5^ Division of Pediatric Hematology‐Oncology Department of Pediatrics Moores Cancer Center UC San Diego School of Medicine La Jolla California USA; ^6^ Levine Cancer Institute Charlotte North Carolina USA; ^7^ Department of Genetics Cell Biology, and Anatomy University of Nebraska Medical Centre Omaha Nebraska USA; ^8^ Division of Clinical Microbiology Department of Laboratory Medicine Karolinska Institute Stockholm Sweden

Dear Editor,

We demonstrated that SF2523, a dual small molecule inhibitor of PI3K‐α/mTOR/BRD4 pathways, can inhibit the replication of SARS‐CoV‐2 and its emerging variants of concern (VOCs), including Delta and Omicron. Further, we also found that SF2523 acts synergistically with remdesivir (RDV) and MU‐UNMC‐2 (a small molecule entry inhibitor of SARS‐CoV‐2).^1^ The ongoing COVID‐19 pandemic due to the emergence of a novel coronavirus SARS‐CoV‐2 remains a significant health concern globally. Several vaccine candidates and anti‐virals received emergency use authorization. However, these vaccines/anti‐virals safety, efficacy and durability remain unknown, especially for the individuals with comorbid conditions, and VOCs, such as Delta, Omicron, BA.2 and Deltacron, which have evolved mutations in the receptor‐binding domain of SARS‐CoV‐2 spike protein may even evade antibodies induced by vaccines or natural infection.^2^ Similarly, recently FDA‐approved Molnupiravir and PAXLOVID remain sensitive towards VOCs.^3^ The in‐depth understanding of the molecular mechanism of the SARS‐CoV‐2 lifecycle revealed the interaction of several host factors with viral proteins essential for the reproduction of progeny viruses, such as bromodomain, containing extra‐terminal domain proteins (BETs), and the mTOR pathway.^4^

Recent studies identified 67 potential interactions between host and viral proteins essential for the SARS‐CoV‐2 lifecycle, like BRD2/BRD4 with the E protein of SARS‐CoV‐2,^4^ and suggested that BRD2 inhibition downregulates ACE2 expression, blocks the entry of SARS‐CoV‐2 into host cells and controls hyperactive immune response in COVID‐19 patients through downregulation Interferon stimulated genes (ISGs).^5^ Targeted therapies that exploit host–virus interaction are likely to be least impacted by the VOCs of SARS‐CoV‐2 and are expected to produce more robust, durable treatment options. Therefore, we tested the anti‐viral potential of SF2523 against the wild‐type SARS‐CoV‐2 and VOCs. In UNCN1T (a bronchial epithelial cell line), Vero STAT1 KO and Calu‐3 cells, the CC_50_ value of SF2523 is above 100 μM (Figure 1A,B **;** Figure S2A). SF2523 showed potent anti‐viral activity with an IC_50_ of 1.52 μM (RDV IC_50_ of 1.06 μM) in UNCN1T cells (Figure 1C) and 1.02 μM (RDV IC_50_ of 1.03 μM) in Vero STAT1 KO cells (Figure 1D) at 24 hpi, respectively. At 48 hpi in UNCN1T cells, SF2523 has an IC_50_ of 1.58 μM (RDV IC_50_ of 2.75 μM) and in Vero STAT1 KO cells has an IC_50_ of 3.22 μM (RDV IC_50_ of .76 μM), respectively (Figure S1A; 1B). Similarly, in Calu‐3 cells, SF2523 has an anti‐viral activity with an IC_50_ of 2.08, 4.03, 0.86 and 4.03 μM against the Delta variant (linage: B.1.617.2; Figure 2A), the Omicron variant (linage: B.1.1.529; Figure 2B), the South African variant (linage: B.1.351; Figure 2C) and the Scotland variant (linage: B.1.222; Figure 2D), respectively. Since BRD4 promotes lung tissue fibrosis through an increase in the expression of pro‐fibrotic genes, and the majority of severely ill COVID‐19 patients suffer from pulmonary fibrosis,^6^ the inclusion of BRD4 inhibitor SF2523 in COVID‐19 therapy will benefit patients to recover from lung fibrosis. This is further supported by a recent study showing that BET protein inhibition blocks cardiac irregularities and SARS‐CoV‐2 infection^7^ and may be useful to controlling the long COVID‐19 effects recently seen in many recovered patients.

**FIGURE 1:**
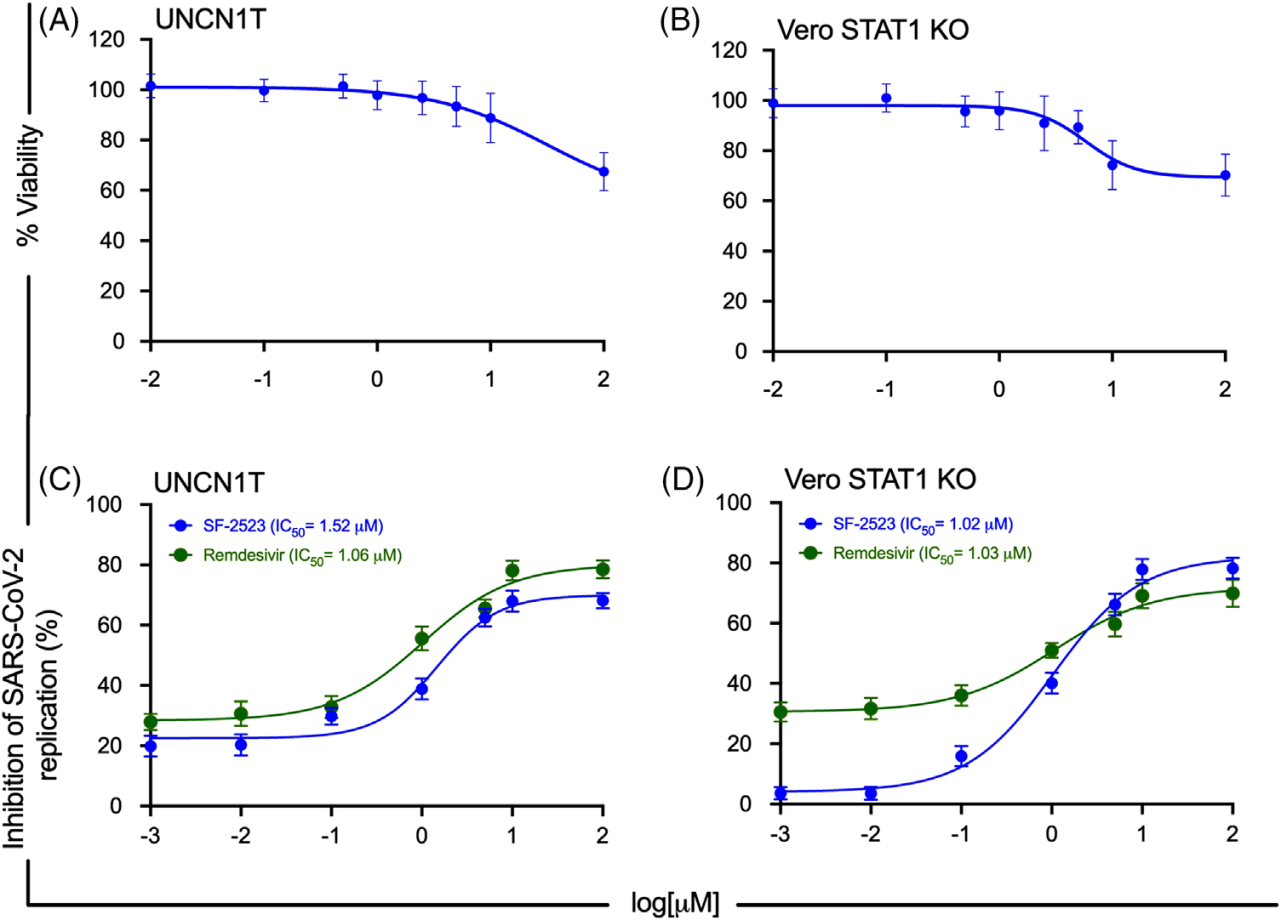
Cytotoxicity and wild‐type SARS‐CoV‐2 replication inhibition dose–response curves of SF2523 in UNCN1T and Vero STAT1 KO cells. (A, B) Cytotoxicity of SF2523 in UNCN1T and Vero STAT1 KO cells was measured by MTT assay. The cells were treated with increasing concentration of SF2523 (0.001–100 μM) and incubated at 37°C in a humidified 5% CO_2_ incubator. Seventy‐two hours post‐treatment, 20 μl of MTT substrate (5 mg/ml) was added to each well and incubated for 4 additional hours at 37°C in the dark. Then, the culture media was carefully removed, and blue formazan crystals were dissolved in 200 μl of DMSO, and the purple colour was read at 595 nm with a reference filter of 620 nm. In both the cells, SF2523 has a CC_50_ value above 100 μM. (C, D) UNCN1T and Vero STAT1 knockout cells (20,000 cells/well) were seeded in 96‐well plates 24 h before infection. Different concentrations of SF2523 and remdesivir (100, 10, 5, 1, 0.1, 0.01 and 0.001 μM) were added to the cells 2 h before infection. The cells were infected with 0.1 MOI of SARS‐CoV‐2 isolate USA‐WI1/2020 (BEI Cat # NR52384). Culture supernatant was collected 24 h post‐infection. The SARS‐CoV‐2 viral load was quantified in the culture supernatant using RT‐qPCR with primer probes targeting E gene of SARS‐CoV‐2 using PrimeDirect Probe RT‐qPCR Mix (TaKaRa Bio USA, Inc) and Applied Biosystems QuantStudio3 real‐time PCR system (Applied Biosystems, Waltham, MA, USA) as per the manufacturer's instructions. The SARS‐CoV‐2 genome equivalent copies were calculated using quantitative PCR (qPCR) control RNA from heat‐inactivated SARS‐CoV‐2, isolate USA‐WA1/2020 (BEI, Catalog# NR‐52347). The percentage inhibition of SARS‐CoV‐2 replication in SF2523 and remdesivir‐treated wells was calculated with respect to viral concentration in positive control wells treated with DMSO (considered 0% inhibition) and negative control wells (uninfected cells). SF2523 (in blue) and remdesivir (in green) IC_50_ values were calculated using four‐parameter variable slope sigmoidal dose–response models using Graph Pad Prism 8.0 software; [CC_50_: drug concentration that is required to reduce cell viability by 50%; IC_50_: drug concentration that is required to reduce the viral replication by 50%]

**FIGURE 2:**
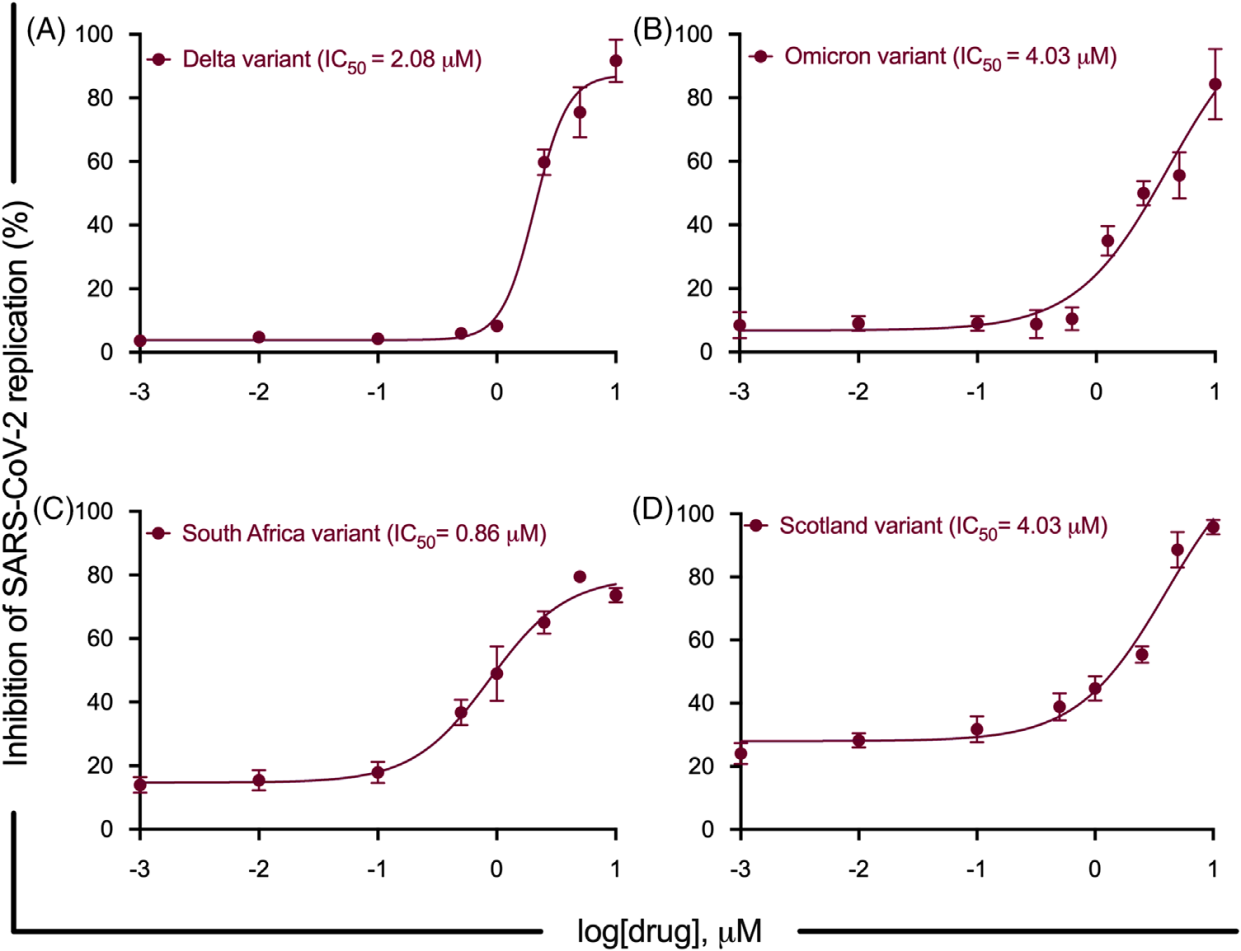
SARS‐CoV‐2 mutant variants replication inhibition dose–response curves of SF2523 in Calu‐3 cells. Calu‐3 cells (20, 000 cells/well) were seeded in a 96‐well plate 72 h before infection. Different concentrations of SF2523 (0.001–10 μM) were added to the cells 2 h before infection. The cells were infected with 0.5 MOI of different SARS‐CoV‐2 mutant variants. Culture supernatant was collected at 24 h post‐infection. The SARS‐CoV‐2 viral load was quantified in culture supernatant using RT‐QPCR with primer probes targeting E gene of SARS‐CoV‐2 using PrimeDirect Probe RT‐qPCR Mix (TaKaRa Bio USA, Inc) and Applied Biosystems QuantStudio3 real‐time PCR system (Applied Biosystems) as per the manufacturer's instructions. The SARS‐CoV‐2 genome equivalent copies were calculated using quantitative PCR (qPCR) control RNA from heat‐inactivated SARS‐CoV‐2, isolate USA‐WA1/2020 (BEI, Cat# NR‐52347). The percentage inhibition of SARS‐CoV‐2 replication in SF2523‐treated wells was calculated with respect to viral concentration in positive control wells treated with DMSO (considered 0% inhibition) and negative control wells (uninfected cells). SF2523 IC_50_ values were calculated using four‐parameter variable slope sigmoidal dose–response models using Graph Pad Prism 8.0 software. (A) SF2523 dose–response curve by percentage inhibition of SARS‐CoV‐2 replication at 24 hpi in Calu‐3 cells infected with Delta variant (linage: B.1.617.2) (BEI, Cat # NR‐55672) with indicated drug concentrations; (B) with Omicron variant (linage: B.1.1.529)(BEI, Cat # NR56475) with indicated drug concentrations; (C) with South Africa variant (linage: B.1.351) with indicated drug concentrations and (D) with Scotland variant (linage: B.1.222) with indicated drug concentrations; [CC_50_: drug concentration that is required to reduce cell viability by 50%; IC_50_: drug concentration that is required to reduce the viral replication by 50%]

Combining multiple drugs with different modes of action has synergistic effects and has been used as anti‐virals. Therefore, we evaluated a series of fixed‐dose combinations of SF2523 and RDV or MU‐UNMC‐2.^1^ The combined doses of SF2523/RDV and SF2523/MU‐UNMC‐2 have CC50 values above 100 μM (Figure S2B and C). In UNCN1T cells at 24 hpi, when a fixed dose of 0.1 μM SF2523 is combined with different dosages of RDV, it has an IC_50_ value of 0.62 μM (Figure 3A) that is lower than individual IC_50_ of both the compounds. The dose–response percent inhibition matrix of single and combination treatment of SF2523/RDV and SF2523/MU‐UNMC‐2 is described in Figure 3B and E. The 3D interaction landscape of SF2523/RDV and SF2523/MU‐UNMC‐2 was computed based on Loewe additive model using SynergyFinder v.2 (Figure 3C and F). The synergistic/antagonistic effects of drug combinations are reconfirmed using Chou and Talalay combination index (CI) theorem^8^ [CI < 1: synergism; = 1: additive effect; > 1: antagonism]. Using CompuSyn, we computed CI values of SF2523/RDV as CI < 1 and CI of SF2523/MU‐UNMC‐2 as 0.129, confirming their synergistic effect. For SF2523/RDV, we obtained a dose reduction index (DRI) of 25.33 and 3.75 for SF2523 and RDV, and for SF2523/MU‐UNMC‐2, we obtained the DRI of 23.03 and 11.56 for SF2523 and MU‐UNMC‐2, respectively. This synergistic effect of SF2523 with RDV or MU‐UNMC‐2 is expected in a favourable shift in the plasma Cmax/EC_90_ ratio. Next, using NMR titration experiments by labelling ^15^N‐labelled BRD4 domains, we showed that RDV is not able to bind to either BRD4‐BD1, BRD4‐BD2 or the ET domain of BRD4, as no significant chemical shift perturbations were observed in ^1^H,^15^N heteronuclear single quantum coherence spectra of these domains (Figure 4). This confirms that RDV does not alter the potency of SF2523 and does not interfere with the binding of SF2523 to BRD4.

**FIGURE 3:**
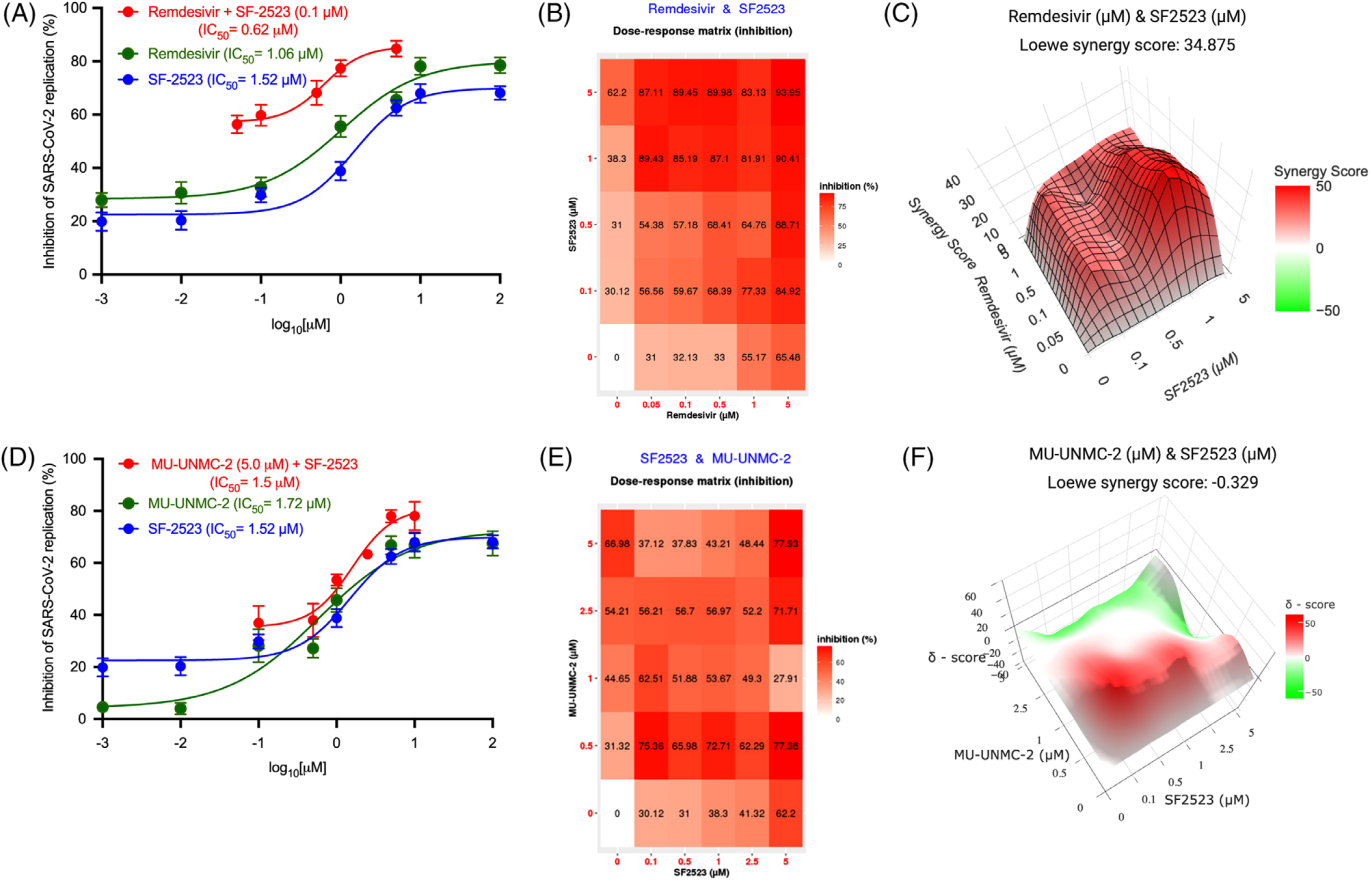
SF2523/remdesivir and SF2523/MU‐UNMC‐2 act synergistically to block the replication of SARS‐CoV‐2. Combinational anti‐viral effect of SF2523/remdesivir and SF2523/MU‐UNMC‐2 against SARS‐CoV‐2 was tested in different fixed‐dose combinations in SARS‐CoV‐2‐infected UNCN1T cells. The UNCN1T cells were seeded in 96‐well plates (20, 000 cells/wells) 24 h before infection. Two hours before infection, the cells were treated with different combination doses of SF2523 and remdesivir or SF2523 and MU‐UNMC‐2 and then infected with 0.1 MOI of SARS‐CoV‐2 isolate USA‐WI1/2020. Twenty‐four hours post‐infection, culture supernatant was collected, and SARS‐CoV‐2 viral load was quantified using RT‐qPCR. The percentage inhibition of SARS‐CoV‐2 replication in the culture supernatant by different combined doses of SF2523 and remdesivir or SF2523 and MU‐UNMC‐2 was determined with respect to viral concentration in positive control wells that were treated with DMSO (considered 0% inhibition) and negative control wells (uninfected cells). Dose–response percent inhibition matrix of single and combined treatment of SF2523 and remdesivir or SF2523 and MU‐UNMC‐2 in SARS‐CoV‐2‐infected UNCN1T cells 24 h post‐infection, and 3D interaction landscape was calculated based on Loewe additive model using SynergyFinder v.2. (A) Dose–response curve of remdesivir (green: IC_50_ = 1.06 μM), SF2523 (blue: IC_50_ = 1.52 μM), and remdesivir with a fixed‐dose combination of SF2523 (0.1 μM) (red: IC_50_ = 0.625 μM) by percentage inhibition of SARS‐CoV‐2 replication at 24 hpi in UNCN1T cells. (B) Dose–response percent inhibition matrix of single and combined treatment of SF2523 and remdesivir in SARS‐CoV‐2‐infected UNCN1T cells at 24 hpi. (C) 3D interaction landscape between SF2523 and remdesivir calculated based on Loewe additive model using SynergyFinder v.2 in SARS‐CoV‐2‐infected UNCN1T cells at 24 hpi (Loewe synergy score 34.87). (D) Dose–response curve of MU‐UNMC‐2 (green: IC_50_ = 1.72 μM), SF2523 (blue: IC_50_ = 1.52 μM) and SF2523 with a fixed‐dose combination of MU‐UNMC‐2 (5 μM) (red: IC_50_ = 1.5 μM) by percentage inhibition of SARS‐CoV‐2 replication at 24 hpi in UNCN1T cells. (E) Dose–response percent inhibition matrix of single and combined treatment of SF2523 and MU‐UNMC‐2 in SARS‐CoV‐2‐infected UNCN1T cells at 24 hpi. (F) 3D interaction landscape between SF2523 and MU‐UNMC‐2 calculated based on Loewe additive model using SynergyFinder v.2 in SARS‐CoV‐2‐infected UNCN1T cells at 24 hpi (Loewe synergy score –0.329)

**FIGURE 4:**
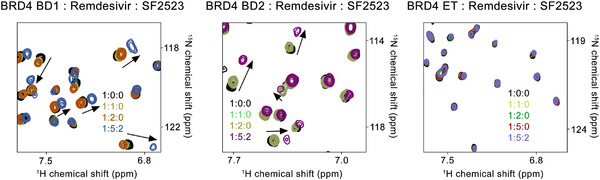
Remdesivir does not interfere with the interaction between SF2523 and BRD4. BRD4 ^15^N‐labelled BD1 (amino acids 43–180), BD2 (amino acids 342–460) and ET domain (amino acids 600–700) were expressed in *Escherichia coli*. Superimposed ^1^H,^15^N heteronuclear single quantum coherence (HSQC) spectra of uniformly ^15^N‐labelled BD1 (left), BD2 (middle) and ET (right) domains of BRD4 with the indicated ligands added in a stepwise manner. Two‐dimensional ^1^H,^15^N HSQC spectra were acquired on a 600 MHz Varian spectrometer fitted with a cryogenic probe at 298 K. Spectra were processed with NMRPipe. The spectra are colour‐coded according to the protein: ligand ratios. Small resonance changes are due to weak binding of DMSO, which was confirmed by titrating DMSO alone to separate samples of these domains (data not shown)

It is documented that SARS‐CoV‐2 infection in mammalian cells inhibits autophagy through various mechanisms, including the activation of autophagy inhibitory proteins (AKT1 and SKP2) and inhibition of proteins involved in autophagy initiation (AMPK, TSC2, Unc‐51 Like Autophagy Activating Kinase 1 and Beclin 1), autophagosome formation (VPS34) and in membrane tethering and fusion of autophagosomes to endolysosomes (ATG14)^9^ and SARS‐CoV‐2, and its transmembrane protein ORF3a inhibits autophagy. Activating autophagy using small molecules that target PI3Kinase/Akt pathway or stabilize Beclin inhibits SARS‐CoV‐2 replication.^9^ Our studies suggest that SF2523 may be modulating the autophagy mechanisms (Figure S3) to limit SARS‐CoV‐2 infection similar to previously reported HIV‐infected macrophages restrict infection through autophagy induction,^10^ although this warrants further in‐depth investigation.

In conclusion, we demonstrated that SF2523 could inhibit the replication of SARS‐CoV‐2 and its VOCs. Further, we found a synergistic effect of SF2523 with RDV or MU‐UNMC‐2 in a wide dose range. Therefore, we conclude that SF2523 (a PI3K‐α/mTOR/BRD4 inhibitor) alone or combined with other anti‐virials represents a future therapeutic approach to prevent the severe disease associated with SARS‐CoV‐2 and its VOCs.

## Supporting information


**Fig S1. Wild type SARS‐CoV‐2 replication inhibition dose‐response curves of SF2523 in UNCN1T and Vero STAT1 KO cells. (A, B)**. UNCN1T and Vero STAT1 knockout cells (20,000 cells/well) were seeded in 96‐well plates 24 hours before infection. Different concentrations of SF2523 and remdesivir (100 μM, 10 μM, 5 μM, 1 μM, 0.1 μM, 0.01 μM and 0.001 μM) were added to the cells 2 hours before infection. The cells were infected with 0.1 MOI of SARS‐CoV‐2 isolate USA‐WI1/2020. Culture supernatant was collected at 48 hrs post‐infection. The SARS‐CoV‐2 viral load was quantified in the culture supernatant using RT‐QPCR with primer probes targeting E gene of SARS‐CoV‐2 using PrimeDirect Probe RT‐qPCR Mix (TaKaRa Bio USA, Inc) and Applied Biosystems QuantStudio3 real‐time PCR system (Applied Biosystems, Waltham, MA, USA) per the manufacturer's instructions. The SARS‐CoV‐2 genome equivalent copies were calculated using quantitative PCR (qPCR) control RNA from heat‐inactivated SARS‐CoV‐2, isolate USA‐WA1/2020 (BEI, Catalog# NR‐52347). The percentage inhibition of SARS‐CoV‐2 replication in SF2523 and remdesivir treated wells was calculated with respect to virus concentration in positive control wells treated with DMSO (considered 0% inhibition) and negative control wells (uninfected cells). SF2523 (in blue) and remdesivir (in green) IC_50_ values were calculated using four‐parameter variable slope sigmoidal dose‐response models using Graph Pad Prism 8.0 software; [CC_50_: Drug concentration that required to reduces cell viability by 50%; IC_50_: Drug concentration that required to reduce the viral replication by 50%].
**Fig S2. Cytotoxicity of SF2523 in Calu‐3 cells (A) and combination of SF2523/RDV and SF2523/MU‐UNMC‐2 in UNCN1T cells (B and C) cells was measured by MTT assay**. The cells were treated with increasing concentration of SF2523, SF2523/RDV and SF2523/MU‐UNMC‐2 (0.001 to 100 μM) and incubated at 37° C in a humidified 5% CO2 incubator. After 72‐hour post‐treatment, 20 μL of MTT substrate (5 mg/mL) was added to each well and incubated for 4 additional hours at 37° C in the dark. Then the culture media was carefully removed, and blue formazan crystals were dissolved in 200 °l of DMSO, and the purple color was read at 595 nm with a reference filter of 620 nm. In respective cells, SF2523, SF2523/RDV and SF2523/MU‐UNMC‐2 has a CC_50_ value above 100 °M.
**Fig S3. SF2523 may induces autophagy in SARS‐CoV‐2 infected Vero cells**. The cells were treated with the indicated concentration of SF2523 and 5 mM 3‐MA and infected with SARS‐CoV‐2. After 24h, cells were harvested, cell lysate prepared, total protein content quantified, and immunoblotting was performed. The upper and lower panels are the representative immunoblots of p62 and LC3B‐I//II. Actin was used as a loading control. The upper and lower bar graphs show densitometric analysis of p62 and LC3B‐II immunoblots, respectively normalized with actin. Data is expressed as mean ± SEM derived from two experiments.
