# Supplementary material for: PI3K‐α/mTOR/BRD4 inhibitor alone or in combination with other anti‐virals blocks replication of SARS‐CoV‐2 and its variants of concern including Delta and Omicron
Source: Clin Transl Med. 2022 Apr 7;12(4):e806. doi: 10.1002/ctm2.806 (PMC8989379; doi:10.1002/ctm2.806)
Supplement: Supplementary file 1 — Fig S1. Wild type SARS‐CoV‐2 replication inhibition dose‐response curves of SF2523 in UNCN1T and Vero STAT1 KO cells. (A, B). UNCN1T and Vero STAT1 knockout cells (20,000 cells/well) were seeded in 96‐well plates 24 hours before infection. Different concentrations of SF2523 and remdesivir (100 μM, 10 μM, 5 μM, 1 μM, 0.1 μM, 0.01 μM and 0.001 μM) were added to the cells 2 hours before infection. The cells were infected with 0.1 MOI of SARS‐CoV‐2 isolate USA‐WI1/2020. Culture supernatant was collected at 48 hrs post‐infection. The SARS‐CoV‐2 viral load was quantified in the culture supernatant using RT‐QPCR with primer probes targeting E gene of SARS‐CoV‐2 using PrimeDirect Probe RT‐qPCR Mix (TaKaRa Bio USA, Inc) and Applied Biosystems QuantStudio3 real‐time PCR system (Applied Biosystems, Waltham, MA, USA) per the manufacturer's instructions. The SARS‐CoV‐2 genome equivalent copies were calculated using quantitative PCR (qPCR) control RNA from heat‐inactivated SARS‐CoV‐2, isolate USA‐WA1/2020 (BEI, Catalog# NR‐52347). The percentage inhibition of SARS‐CoV‐2 replication in SF2523 and remdesivir treated wells was calculated with respect to virus concentration in positive control wells treated with DMSO (considered 0% inhibition) and negative control wells (uninfected cells). SF2523 (in blue) and remdesivir (in green) IC50 values were calculated using four‐parameter variable slope sigmoidal dose‐response models using Graph Pad Prism 8.0 software; [CC50: Drug concentration that required to reduces cell viability by 50%; IC50: Drug concentration that required to reduce the viral replication by 50%]. Fig S2. Cytotoxicity of SF2523 in Calu‐3 cells (A) and combination of SF2523/RDV and SF2523/MU‐UNMC‐2 in UNCN1T cells (B and C) cells was measured by MTT assay. The cells were treated with increasing concentration of SF2523, SF2523/RDV and SF2523/MU‐UNMC‐2 (0.001 to 100 μM) and incubated at 37° C in a humidified 5% CO2 incubator. After 72‐hour post‐treatment, 20 μL of MTT subs [file CTM2-12-e806-s001.pdf]

## Supporting Information

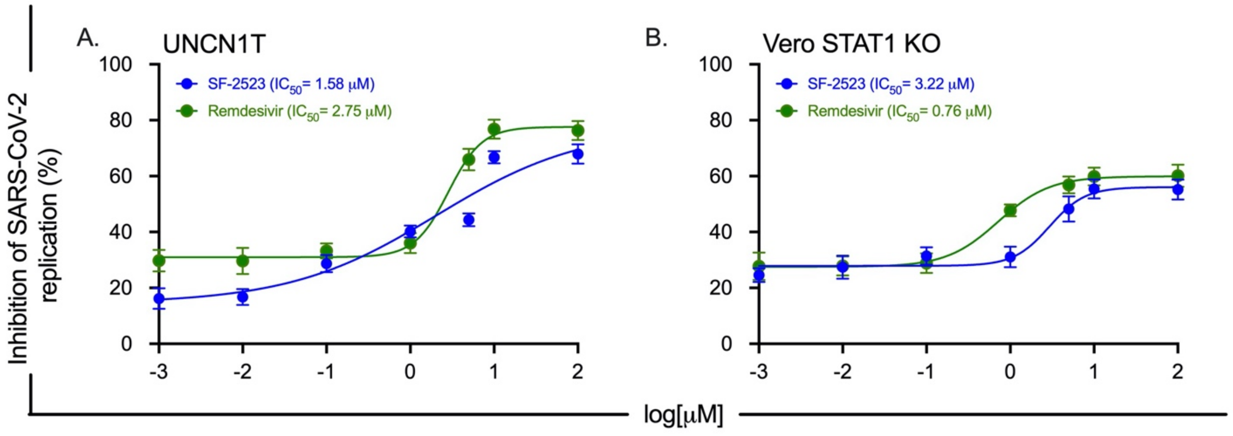


**Fig S1. Wild type SARS-CoV-2 replication inhibition dose-response curves of SF2523 in UNCN1T and Vero STAT1 KO cells. (A, B).** UNCN1T and Vero STAT1 knockout cells (20,000 cells/well) were seeded in 96-well plates 24 hours before infection. Different concentrations of SF2523 and remdesivir (100 μM, 10 μM, 5 μM, 1 μM, 0.1 μM, 0.01 μM and 0.001 μM) were added to the cells 2 hours before infection. The cells were infected with 0.1 MOI of SARS-CoV-2 isolate USA-WI1/2020. Culture supernatant was collected at 48 hrs post-infection. The SARS-CoV-2 viral load was quantified in the culture supernatant using RT-QPCR with primer probes targeting E gene of SARS-CoV-2 using PrimeDirect Probe RT-qPCR Mix (TaKaRa Bio USA, Inc) and Applied Biosystems QuantStudio3 real-time PCR system (Applied Biosystems, Waltham, MA, USA) per the manufacturer's instructions. The SARS-CoV-2 genome equivalent copies were calculated using quantitative PCR (qPCR) control RNA from heat-inactivated SARS-CoV-2, isolate USA-WA1/2020 (BEI, Catalog# NR-52347). The percentage inhibition of SARS-CoV-2 replication in SF2523 and remdesivir treated wells was calculated with respect to virus concentration in positive control wells treated with DMSO (considered 0% inhibition) and negative control wells (uninfected cells). SF2523 (in blue) and remdesivir (in green) $IC_{50}$ values were calculated using four-parameter variable slope sigmoidal dose-response models using Graph Pad Prism 8.0 software; [$CC_{50}$: Drug concentration that required to reduces cell viability by 50%; $IC_{50}$: Drug concentration that required to reduce the viral replication by 50%].

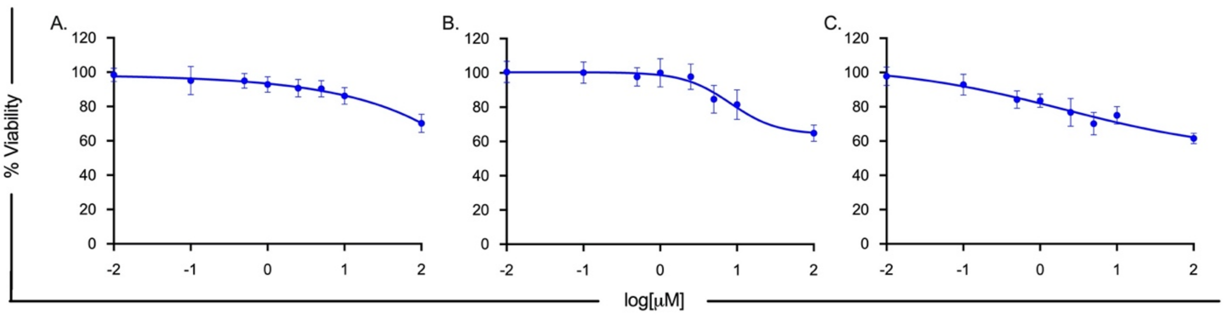


**Fig S2. Cytotoxicity of SF2523 in Calu-3 cells (A) and combination of SF2523/RDV and SF2523/MU-UNMC-2 in UNCN1T cells (B and C) cells was measured by MTT assay**. The cells were treated with increasing concentration of SF2523, SF2523/RDV and SF2523/MU-UNMC-2 (0.001 to 100 µM) and incubated at 37º C in a humidified 5% $CO_2$ incubator. After 72-hour post-treatment, 20 µL of MTT substrate (5 mg/mL) was added to each well and incubated for 4 additional hours at 37º C in the dark. Then the culture media was carefully removed, and blue formazan crystals were dissolved in 200 µl of DMSO, and the purple color was read at 595 nm with a reference filter of 620 nm. In respective cells, SF2523, SF2523/RDV and SF2523/MU-UNMC-2 has a $CC_{50}$ value above 100 µM.

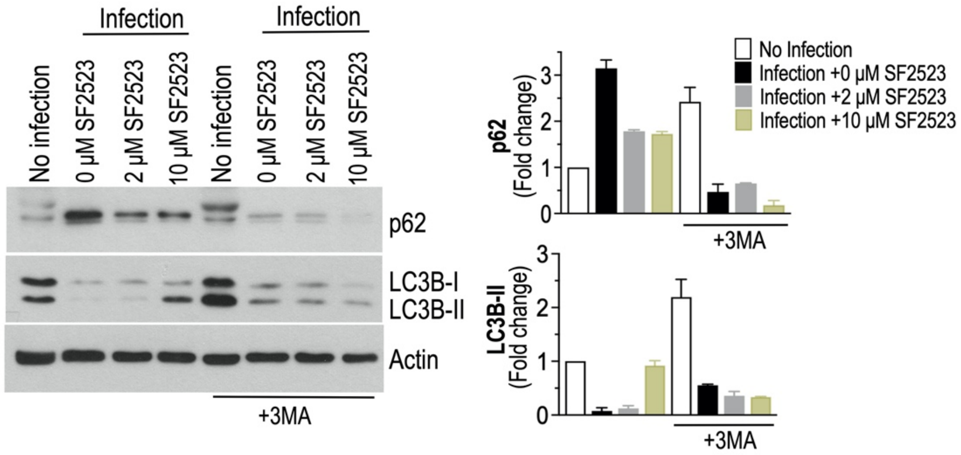


**Fig S3. SF2523 may induces autophagy in SARS-CoV-2 infected Vero cells.** The cells were treated with the indicated concentration of SF2523 and 5 mM 3-MA and infected with SARS-CoV-2. After 24h, cells were harvested, cell lysate prepared, total protein content quantified, and immunoblotting was performed. The upper and lower panels are the representative immunoblots of p62 and LC3B-I//II. Actin was used as a loading control. The upper and lower bar graphs show densitometric analysis of p62 and LC3B-II immunoblots, respectively normalized with actin. Data is expressed as mean ± SEM derived from two experiments.
